# Alterations in Baroreflex Sensitivity and Blood Pressure Variability Following Sport-Related Concussion

**DOI:** 10.3390/life12091400

**Published:** 2022-09-08

**Authors:** Chase J. Ellingson, Jyotpal Singh, Cody A. Ellingson, Luke W. Sirant, Gregory P. Krätzig, Kim D. Dorsch, Jaroslaw Piskorski, J. Patrick Neary

**Affiliations:** 1 Faculty of Kinesiology & Health Studies, University of Regina, 3737 Wascana Parkway, Regina, SK S4S 0A2, Canada; 2 College of Medicine, University of Manitoba, 66 Chancellors Cir, Winnipeg, MB R3T 2N2, Canada; 3 Department of Psychology, University of Regina, 3737 Wascana Parkway, Regina, SK S4S 0A2, Canada; 4 Institute of Physics, University of Zielona Góra, Licealna 9, 65-417 Zielona Góra, Poland

**Keywords:** brain injuries, autonomic nervous system, mild traumatic brain injury, arterial baroreflex, heart rate variability

## Abstract

Current methods to diagnose concussions are subjective and difficult to confirm. A variety of physiological biomarkers have been reported, but with conflicting results. This study assessed heart rate variability (HRV), spontaneous baroreflex sensitivity (BRS), and systolic blood pressure variability (BPV) in concussed athletes. The assessment consisted of a 5-min seated rest followed by a 5-min (0.1 Hz) controlled breathing protocol. Thirty participants completed baseline assessments. The protocol was repeated during the post-injury acute phase (days one to five). Total (*p* = 0.02) and low-frequency (*p* = 0.009) BPV spectral power were significantly decreased during the acute phase of concussion. BRS down-sequence (*p* = 0.036) and up-sequence (*p* = 0.05) were significantly increased in the acute phase of concussion, with a trend towards an increased BRS pooled (*p* = 0.06). Significant decreases in HRV were also found. Acute concussion resulted in altered BRS and BPV dynamics compared to baseline. These findings highlight objective physiological parameters that could aid concussion diagnosis and return-to-play protocols.

## 1. Introduction

The incidence of sport-related concussion (SRC) has increased steadily over the past few decades related to our awareness and ability to monitor the brain non-invasively [1,2,3]. Concussions result from biomechanical forces applied to the body and head, causing significant compressive, tensile, or shear forces within the cranium, leading to neural tissue damage [4]. Sustaining a concussion can initiate a neurometabolic cascade leading to increased physiological stress and permeability of the blood-brain barrier, accompanied by decreases in cerebral blood flow, autoregulation, and parasympathetic activity [5,6]. This may manifest as a state resembling an energy crisis accompanied by impaired neuro-autonomic cardiovascular functioning, neuronal dysfunction, and neuronal inflammation [6,7].

Despite considerable effort, establishing clear diagnostic criteria for concussion remains elusive [8]. This lack of diagnostic criteria has resulted in clinicians having to rely on clinical judgment when diagnosing and managing concussed athletes [9]. Therefore, there is a need to identify objective biomarkers of concussion. Functional magnetic resonance imaging (fMRI), cerebral perfusion pressure, and other noninvasive magnetic resonance imaging techniques have been proposed as potential tools for concussion diagnosis and monitoring [10,11,12]. However, such techniques’ cost and time-intensive nature preclude their widespread application in the clinical setting [10]. While heart rate variability (HRV), which is the measurement of variability in the time intervals between subsequent heartbeats [13], has gained popularity as a potential diagnostic biomarker in concussion research, varying methods and ambiguous results suggest alternative methodologies must be considered [14]. Additionally, it has been recommended that optimal diagnosis of a concussion may be achieved through a multimodal assessment strategy [9,15]. Two additional potential biomarkers of concussion include spontaneous baroreflex sensitivity (BRS) and systolic blood pressure variability (BPV) [16,17].

BRS reflects the RR intervals per unit change in BP [18]. As such, BRS would be directly affected by respiratory rate, which can result in runs of RR intervals along with distinctly opposite fluctuations in systolic blood pressure [19]. Depending on the heart rate run (acceleration or deceleration), these can be further defined as up or down sequences of BRS. Previous research showed decreases in BRS during the acute concussion phase [17]. However, there are no other studies completed with BRS in mind to confirm these findings, and no study controlled for respiration or utilized a within-participant approach. Therefore, the current research is limited, as individual physiology can influence these parameters and comparison with controls rather than participant baselines can affect the results. Furthermore, no study controlled for respiration during acute concussion when assessing BRS, which further results in ambiguous findings. However, BPV is suggested to be influenced by concussion when controlling for respiration, although the research pertaining to BPV is only available in post-concussion syndrome [16]. As such, there are gaps in the literature with respect to the non-standardized control of respiration rate and the utilization of a between-participant analysis.

Biological rhythms in healthy individuals exist in a dynamic, nonlinear environment in which oscillations in such rhythms are frequent [20]. However, during uncoupling between the cardiovascular (CV) and autonomic nervous system (ANS), these oscillations are expected to decrease [20]. Acute brain injury has been demonstrated to reduce CV-ANS variability [17,20,21], and research supports a causal link between the brain and the heart during concussion [22]. Therefore, assessment of the CV-ANS using BRS and BPV, in addition to HRV, has the potential to aid in the diagnosis of concussion.

BRS is a surrogate measure of a negative feedback mechanism that regulates blood pressure (BP), known as the arterial baroreflex [17]. This cardiovascular (CV) reflex regulates BP primarily through the autonomic nervous system (ANS) control of cardiac output and peripheral vascular resistance, increasing or decreasing arterial BP to maintain homeostasis [17,23].

BPV provides valuable insight into the autonomic regulation of the CV system [24,25]. It is thought that the oscillations observed in BPV are influenced by respiration and CV reflexes [24,25]. Random oscillations in rhythmic physiological variables are considered normal in a healthy state [21]. As a result, attenuation of the variability in these oscillations can indicate pathology, suggesting potential CV-ANS dysfunction [21].

Considering that HRV results in the literature are ambiguous [14], we aimed to assess other markers of CV-ANS function in the context of acute SRC. This study aimed to examine the impact of acute SRC on HRV, BRS, and BPV. To address the identified gaps in the literature, we utilized a within-participant approach and controlled for fluctuations in BP and RR intervals by using a controlled breathing protocol. We hypothesized that these parameters would be altered during acute concussion compared to baseline values.

## 2. Materials and Methods

### 2.1. Participants

Baseline physiologic concussion testing was conducted on all varsity-level athletes at the University of Regina, Regina, Canada. To be included in this retrospective observational analysis, it was ensured that all included participants had a valid baseline (BL) assessment and subsequently sustained a diagnosed concussion, as suspected by a certified athletic therapist and confirmed by a team physician. A valid BL assessment was judged by the absence of artifact in the BP or ECG waveform [15]. Participants were instructed to refrain from caffeine for 6 h, exercise for 12 h, and alcohol for 24 h before assessments [26]. An initial acute assessment was completed within five days post-injury. Thirty athletes (age: 20.0 ± 2.0; BMI: 25.1 ± 4.2; 12 female) who sustained a concussion met the inclusion criteria and were included in the HRV analysis.

### 2.2. Data Collection

Participants’ symptoms were recorded using the SCAT 5 symptom evaluation questionnaire [27]. The assessments included a 5-min seated rest period to establish baseline physiology, followed by 5-min of a seated (0.1 Hz) controlled breathing protocol. Given the influence of respiration rate on heart rate (HR), independent of ANS activity [13], it is crucial to control respiration when assessing BRS and BPV. Furthermore, a respiratory frequency of 0.1 Hz elucidates a BP drive with a concurrent HR response [19], eliciting a respiratory-induced baroreflex. Beat-to-beat BP was collected using finger photoplethysmography, with the finger cuff placed around the left middle finger and the height correction unit placed on the participant at the level of the heart (NOVA and Finometer, Finapres Medical Systems BV, Enschede, The Netherlands). In addition, R-R interval data was collected using a three-lead electrocardiogram (ECG) (Lead I configuration). The raw data signals were simultaneously sampled at 1000 Hz and displayed using PowerLab and LabChart software (AD Instruments, Colorado Springs, CO, USA). ANS function, as measured by HRV, exhibits a degree of day-to-day variation [28]. However, this variation is not statistically significant if multiple recordings are collected under similar conditions [28]. For example, ANS activity fluctuates throughout the day as rhythmic circadian changes modulate endogenous physiological mechanisms [29]. Accordingly, all repeat follow-up testing was conducted under identical conditions to the BL assessment and was completed at the same time of day to limit the potential impact of circadian changes.

### 2.3. Data Analysis

The BP and ECG waveforms were visually inspected for the presence of artifact and ectopy. Artifact presence resulted in the removal of the data set. The R-R intervals were analyzed, and power spectral density was estimated using the Lomb method [30]. HRV was assessed using spectral analysis (low-frequency (LF), high-frequency (HF), total power (TP), and LF/HF ratio), time-domain methods (total variability (SDNN)), and statistics derived from Poincaré plots (short-term variability (SD1) and long-term variability (SD2)). The combination of beat-to-beat systolic BP and R-R intervals allowed for the analysis of BRS and BPV using the Ensemble-R software (Elucimed Ltd., Auckland, NZ, USA). BRS was assessed using the parameters BRS-up sequence, BRS-down sequence, and BRS-pooled. Spectral analysis was conducted to assess BPV to obtain LF-, HF-, and TP-BPV. For both HRV and BPV, HF and LF power were measured at 0.15–0.40 Hz and 0.04–0.15 Hz, respectively.

### 2.4. Statistical Analysis

A Wilcoxon signed-rank test was used to compare the paired samples (baseline and acute concussion) with significance set at *p* < 0.05. The data are presented as median (interquartile range).

## 3. Results

Significant HRV decreases were found during acute SRC for SDNN (median = 107.9 ms, Z = −2.0, *p* = 0.045) and SD2 (median = 147.2 ms, Z = −2.1, *p* = 0.039) compared to SDNN (median = 112.5 ms, Z = −2.0, *p* = 0.045) and SD2 (median = 151.6 ms, Z = −2.1, *p* = 0.039) at baseline. Due to artifact in the BP waveform, eight participants were removed, thus resulting in 22 participants included in the BRS and BPV analysis. In the acute visit, BRS-down sequence (median = 16.3 ms/mmHg, Z = −2.1, *p* = 0.036) and BRS-up sequence (median = 26.0 ms/mmHg, Z = −2.0, *p* = 0.05) were significantly increased (*p* < 0.05) compared to the baseline assessment of BRS-down sequence (median = 14.5 ms/mmHg, Z = −2.1, *p* = 0.036) and BRS-up sequence (median = 20.7 ms/mmHg, Z = −2.0, *p* = 0.05). There was a trend towards an increase in BRS-pooled following concussion (*p* = 0.06). The BPV analysis showed significant decreases (*p* < 0.05) in LF (median = 69.4 mmHg^2^, Z = −2.6, *p* = 0.009) and TP (median = 106.0 mmHg^2^, Z = −2.3, *p* = 0.02), at acute injury compared to baseline LF (median = 88.3 mmHg^2^, Z = −2.6, *p* = 0.009) and TP (median = 107.2 mmHg^2^, Z = −2.3, *p* = 0.02). All HRV, BRS, and BPV results comparing the baseline and acute assessments are presented in Table 1.

## 4. Discussion

This study aimed to examine the impact of SRC on HRV, BRS, and BPV. With HRV changes following SRC being previously reported in the literature [5,31], our primary focus was to examine BRS and BPV. To the author’s knowledge, this is the first study using a within-participant approach to investigate the impact of acute concussion on BRS and BPV. As such, this study provides important information concerning BRS and BPV, in addition to HRV, as potential objective physiological measures to assist concussion diagnosis.

Previous research suggests that HRV and BRS are decreased following concussion; however, significant limitations and variability in methodology exist [31]. It has been shown that BRS is depressed in individuals after sustaining a concussion or mild traumatic brain injury (mTBI), suggesting CV-ANS dysfunction [17,20,21,31]. It has been demonstrated that BPV is impaired in post-concussion syndrome and subsequently increased in association with improved symptoms [16]. However, the existing literature on BPV and BRS in concussion fails to use a within-participant analysis, significantly limiting the generalizability and validity of such results. Given the sizeable between-participant variability in autonomic functioning [32], a within-participant analysis can provide more meaningful comparisons. By using a within-participant approach, the BL assessments act as the control group, limiting the potential for erroneous conclusions due to normal between-participant variability in BL values.

Our analysis demonstrates decreased HRV and BPV, along with increases in BRS. Exaggerating the respiratory sinus arrhythmia using a controlled breathing protocol induces fluctuations in the HR responses, thus influencing HRV [19]. Our results showed that at this breathing rate, there were minor HRV response changes due to acute SRC, with the greater compensation occurring through a change in BPV. Although speculative, we suggest that the observed decreases in LF- and TP-BPV and the increases in BRS-down and BRS-up during the controlled breathing protocol represent a compensatory mechanism to maintain adequate cerebral perfusion in the context of the metabolic crisis resulting from a concussion. More research is warranted to test our hypothesis.

Acute SRCs are known to alter cardiovascular indices [5,17,33]. This is the first study to assess BRS and BPV using a within-participant sample while controlling for respiration, thus strengthening the methodology over previous resting condition research. Furthermore, a controlled breathing assessment of BRS and BPV can be easily measured, is time-efficient, and does not exacerbate symptoms in the context of acute SRC. Given this, our findings provide another parameter which can be included for clinical use following acute SRC.

## 5. Conclusions

The findings in this paper have important implications for concussion diagnosis. Our results demonstrated significant alterations in HRV, BRS, and BPV following acute SRC. In addition, using a within-participant analysis, our research provides more methodologically rigorous data than the existing literature. To address our limitations, future research should be aimed at analyzing a larger group of participants and stratifying by sport, sex, and considering a previous history of concussion. These findings would therefore be more generalizable. Finally, follow-up comparisons with BRS, BPV and HRV can provide insights into the recovery period. Comparing these hemodynamic parameters in association with those such as cerebral perfusion pressure [12] can help limit the risk of a secondary injury.

## Figures and Tables

**Table 1:** Heart rate variability (*n* = 30), spontaneous baroreflex sensitivity (*n* = 22), and systolic blood pressure variability (*n* = 22) during the six-breath per minute-controlled breathing protocol.

	Baseline	Acute	Z-Statistic	*p*-Value
LF-HRV (ms^2^)	60.5 (60.2)	61.8 (80.7)	−1.1	0.28
HF-HRV (ms^2^)	4.8 (8.0)	5.5 (7.9)	−1.1	0.26
TP-HRV (ms^2^)	72.7 (69.5)	75.5 (87.9)	−1.1	0.26
LF/HF	12.1 (11.6)	13.3 (6.0)	−0.3	0.77
SDNN * (ms)	112.5 (41.2)	107.9 (46.4)	−2.0	0.045
SD1 (ms)	46.4 (32.4)	43.5 (38.1)	−1.4	0.17
SD2 * (ms)	151.6 (54.3)	147.2 (58.4)	−2.1	0.039
BRS-down * (ms/mmHg)	14.5 (6.2)	16.3 (9.1)	−2.1	0.036
BRS-up * (ms/mmHg)	20.7 (14.4)	26.0 (11.7)	−2.0	0.050
BRS-pooled (ms/mmHg)	17.4 (8.4)	21.3 (10.7)	−1.9	0.060
HF-BPV (mmHg^2^)	4.2 (4.8)	3.8 (4.7)	−1.1	0.250
LF-BPV ** (mmHg^2^)	88.3 (57.5)	69.4 (67.6)	−2.6	0.009
TP-BPV * (mmHg^2^)	107.2 (79.4)	106.0 (82.3)	−2.3	0.020
SCAT Symptoms	95% CI (0,1)	95% CI (4,15)	--	--

* (*p* < 0.05), ** (*p* < 0.01); Values are presented as median (interquartile range). HF = high frequency; LF = low frequency; TP = total power; HRV = heart rate variability; SDNN = total variability; SD1 = short-term variability; SD2 = long-term variability; BRS = spontaneous baroreflex sensitivity; BPV = systolic blood pressure variability.

## Data Availability

The datasets are available upon request to the corresponding author.
